# Demographic and Sleep Study Factors Influencing Short-Term Adherence to Positive Airway Pressure Therapy in Obstructive Sleep Apnea

**DOI:** 10.3390/jcm14113988

**Published:** 2025-06-05

**Authors:** Ji Ho Choi, Yeji Lee, Sungkyoung Shin, Tae Kyoung Ha, Sooyeon Suh

**Affiliations:** 1 Department of Otorhinolaryngology-Head and Neck Surgery, Soonchunhyang University Bucheon Hospital, Soonchunhyang University College of Medicine, 170, Jomaru-ro, Bucheon 14584, Republic of Korea; 2 Department of Psychology, Sungshin Women’s University, 2, Bomun-ro 34da-gil, Seongbuk-gu, Seoul 02844, Republic of Korea; yeeyezy@gmail.com (Y.L.); sungyb98@gmail.com (S.S.); 3 Honeynaps Research and Development Center, Honeynaps Co., Ltd., 529, Nonhyeon-ro, Gangnam-gu, Seoul 06126, Republic of Korea; sean.ha@honeynaps.com

**Keywords:** obstructive sleep apnea, positive airway pressure, adherence, compliance

## Abstract

**Objective:** Positive airway pressure (PAP) therapy effectively treats obstructive sleep apnea (OSA), yet adherence to the therapy presents significant challenges. This study identifies demographic and sleep study factors that influence short-term adherence to PAP therapy among patients with OSA by comparing data from adherent and non-adherent groups. **Methods:** Patients diagnosed with OSA via polysomnography who commenced PAP therapy after titration were divided into adherent and non-adherent groups. We employed propensity score matching in a 1:1 ratio based on age, gender, and body mass index (BMI), including a total of 150 patients in the analysis. Logistic regression analyses were conducted on all pertinent variables, excluding those with high multicollinearity. Non-significant variables were omitted from the final model, whose performance was evaluated using a receiver operating characteristic (ROC) curve, calculating the area under the curve (AUC). **Results:** Data from 150 participants (mean age 49.56 ± 14.31 years, 79% males, mean BMI 28.96 ± 5.11) were analyzed. Significant predictors of adherence included smoking status (odds ratio [OR] 0.267; 95% confidence interval [CI], 0.116–0.580; *p* = 0.001), Epworth sleepiness scale (OR 1.080; 95% CI, 1.004–1.166; *p* = 0.042), oxygen desaturation index (ODI) during titration (OR 0.906; 95% CI, 0.829–0.975, *p* = 0.015), and optimal PAP levels (OR 1.240; 95% CI, 1.007–1.119; *p* = 0.029). The ROC curve analysis indicated an AUC of 0.765, confirming the model’s effectiveness in distinguishing between adherent and non-adherent patients. **Conclusions:** Adherence is negatively affected by smoking, whereas higher daytime sleepiness, optimal PAP levels, and a lower ODI during titration are associated with better adherence, underscoring the importance of personalized treatment approaches.

## 1. Introduction

Obstructive sleep apnea (OSA) is increasingly recognized as a chronic condition requiring long-term management, not simply as a sleep disorder [1]. Currently, a variety of treatment options are available for OSA, including positive airway pressure (PAP) therapy, mandibular advancement devices, surgical interventions, positional therapy, and weight management [1]. Each of these modalities has its own advantages and limitations, and the choice of treatment is typically guided by a comprehensive assessment of polysomnography results, anatomical considerations, and the patient’s treatment preferences [1]. PAP therapy is the recommended primary treatment for OSA [2]. OSA occurs due to repetitive narrowing or obstruction of the upper airway during sleep [3], and PAP therapy maintains the airway open through a mask, providing a continuous stream of air [4]. This mechanism, known as “pneumatic splinting,” allows patients to breathe normally during sleep.

Numerous studies have established PAP therapy as a highly effective method for managing OSA and enhancing the sleep-related quality of life of patients [5,6,7,8,9,10]. PAP therapy significantly reduces the frequency of breathing disturbances during sleep, as evidenced by a lower apnea–hypopnea index (AHI; <10 in most cases). This reduction leads to an improved sleep structure, notably, an increased proportion of deep sleep (N3 sleep) and enhanced sleep quality [5,6]. Additionally, it alleviates both objective and subjective daytime sleepiness, thereby reducing the risk of traffic accidents [5,6,7]. PAP therapy also contributes to stable blood pressure management in OSA patients with hypertension, reduces inflammatory responses and blood coagulation, and lowers the risk of major cardiovascular events, such as stroke and cardiovascular death [5,6,8,9]. Moreover, it plays a crucial role in reducing the risk of and managing metabolic and endocrine disorders, such as diabetes [10]. Furthermore, PAP therapy positively impacts the patient’s health and improves the sleep quality of their bed partner, as evidenced by reported enhancements in sleep duration and quality of life measures [11,12].

Despite the well-established benefits of PAP therapy, patient adherence continues to be a significant challenge. The demographic factors influencing adherence to PAP therapy continue to be debated, especially the roles of age and gender [13,14]. However, studies indicate that African Americans generally exhibit lower adherence rates than Caucasians [15]. Regarding socioeconomic factors, a higher socioeconomic status is associated with better adherence [16]. From a psychological perspective, higher self-efficacy—defined as a person’s belief in their ability to perform tasks—prior to initiating therapy has been reported to increase adherence [17]. More severe cases of OSA are generally associated with higher adherence rates [18]. Furthermore, explaining the disease using polysomnography results can improve adherence [19]. Additional factors such as the early selection of a suitable mask, a history of nasal or pharyngeal surgery, and telemonitoring care have also been identified as potential contributors to enhanced adherence to PAP therapy [20,21,22,23].

Cognitive impairment is closely linked to OSA, with a particularly high prevalence among patients with mild cognitive impairment and Alzheimer’s disease. PAP therapy has been shown to alleviate cognitive decline in these populations, with adherence rates of 35.6% to 73%, comparable to the general population [24]. Caregiver support plays a crucial role in promoting adherence among cognitively impaired individuals. Repeated arousals and sleep fragmentation contribute to cognitive decline and increase dementia risk, underscoring the importance of early PAP intervention [25]. Emotional factors such as anxiety, depression, and claustrophobia can significantly reduce PAP adherence, with claustrophobia more than doubling the risk of non-adherence [26]. Psychological assessments may be useful in predicting adherence, as adherent patients often report better sleep quality, lower daytime sleepiness, and lower psychological distress [27]. Social support, including marital status and spousal involvement, has been associated with higher adherence rates [28]. Digital platforms that provide real-time feedback and self-management tools can enhance early compliance by improving motivation and delivering personalized information [29].

According to various studies, adherence rates to PAP therapy range between approximately 29% and 83% [30,31]. Therapeutic responses to PAP therapy vary among patients with OSA, and adherence may decline due to multiple factors. It is important to understand the factors that influence short-term adherence to enhance patient outcomes. Consequently, this study aims to identify factors that affect short-term adherence to PAP therapy in patients with OSA. It involves comparing and analyzing demographic data and sleep study results between adherence and non-adherence groups.

## 2. Materials and Methods

### 2.1. Subjects

The present study was reviewed by and received ethical approval from the Institutional Review Board (IRB) of Soonchunhyang University Bucheon Hospital (IRB No. 2024-12-018). A total of 150 subjects, comprising adherence group (*n* = 75) and non-adherence group (*n* = 75), were included in this investigation. The initial selection of the non-adherent group adhered to the inclusion criteria. To minimize confounding and balance key characteristics between groups, a 1:1 matching procedure based on an approximately 5:1 ratio of adherent to non-adherent participants was employed. Propensity score matching was applied, equally distributing 75 patients in each group by age, gender, and body mass index (BMI). Inclusion criteria for the non-adherence group included the following: (1) adults aged 20 years and above; (2) subjects diagnosed with OSA according to standard polysomnography, requiring an AHI ≥ 5 with clinically suspected symptoms, or an AHI ≥ 15 regardless of symptoms; (3) patients consenting to and prescribed PAP therapy for OSA; and (4) participants who did not meet the PAP adherence criteria [32]. This clinical study was based on a retrospective chart review of patients who underwent PAP titration from March 2020 to October 2023. PAP adherence criteria were defined according to Korean reimbursement standards, necessitating ≥ 4 h of nightly PAP use on ≥70% of nights within a continuous 30-day span during the initial 90 days [33].

### 2.2. Demographic Data

Data collection encompassed demographic information such as age, gender, BMI, and smoking status, as well as subjective assessments of daytime sleepiness and sleep quality. These assessments used the Epworth sleepiness scale (ESS) and the Pittsburgh sleep quality index, respectively, and were obtained through patient questionnaires prior to the polysomnographic evaluation.

### 2.3. Sleep Study Data

Each subject underwent a comprehensive physical examination and completed overnight polysomnography in a calm, dark environment with controlled ambient temperature. Polysomnography monitored a range of parameters including electrooculography, electroencephalography, electrocardiography, chin electromyography, nasal and oral airflow (measured through a thermistor and nasal pressure sensor), chest and abdominal movements (utilizing inductance plethysmography), oxygen saturation (via pulse oximetry), bilateral anterior tibialis electromyography, snoring, and body posture. A sleep technician, using an infrared camera, observed and verified the subjects’ behaviors and sleep postures, and all measurements were conducted using a digitized system (Embla N7000; Natus Medical Inc., San Carlos, CA, USA). The polysomnography analysis highlighted key variables such as total sleep time (TST), sleep efficiency (SE), percent time in each sleep stage, respiratory arousal index, spontaneous arousal index, total arousal index, AHI, supine AHI, respiratory effort-related arousal (RERA) index, respiratory disturbance index (RDI), and oxygen desaturation index (ODI).

All subjects underwent overnight PAP titration accompanied by continuous polysomnographic monitoring, during which a full-night manual titration was performed using the same montage to ascertain the optimal pressure level for PAP treatment. The optimal pressure was defined as the minimum pressure that effectively eliminated respiratory events during sleep, as determined by the final prescribed setting from the PAP titration [34]. The residual event index was calculated using data from the entire PAP titration process to provide a comprehensive assessment of residual respiratory events. The key variables measured in the titration test included TST, SE, the percent time in each sleep stage, total arousal index, AHI, supine AHI, RERA index, RDI, ODI, and optimal pressure. All sleep studies were conducted and manually scored by a sleep technologist in accordance with AASM guidelines and then reviewed by a certified physician [34].

### 2.4. Statistical Analysis

Continuous variables were analyzed using means ± standard deviation, whereas categorical variables were compared using frequency for descriptive statistics. An initial logistic regression model was employed to identify factors associated with adherence to PAP therapy. Here, adherence served the role of the dependent variable, with independent variables encompassing a range of demographic, polysomnographic, and PAP titration data. Variables manifesting high multicollinearity were excluded, as indicated by their variance inflation factor surpassing 10. Results are reported as regression coefficients (B), standard errors, odds ratios (ORs) with 95% confidence intervals (CIs), and *p*-values. Subsequently, variables that did not reach statistical significance were omitted from the initial model. Based on the variables that remained statistically significant, a logistic regression analysis was conducted to develop the final model. Independent variables were standardized to ensure uniformity across different scales, and standardized beta coefficients were calculated to determine the relative contribution of each predictor to adherence. The overall performance of the model was assessed using a receiver operating characteristic (ROC) curve, and the area under the curve (AUC) was computed. Statistical significance was established at *p* < 0.05, with *p* < 0.01 denoting stronger associations. All analyses were performed using R Studio (version 2024.04.2+764; R Foundation for Statistical Computing, Vienna, Austria).

## 3. Results

Demographic and sleep study data from 150 participants (mean age 49.56 ± 14.31 years; 79% male; mean BMI 28.96 ± 5.11) were analyzed. Demographic and sleep study data comparisons between adherence and non-adherence groups are presented in Table 1.

An initial logistic regression model exploring factors associated with short-term adherence is presented in Table 2. Smoking status showed significantly lower adherence when compared to non-smokers (OR 0.301; *p* = 0.009). A lower RERA index was correlated with increased adherence (OR 0.851; *p* = 0.017), and a higher ESS score was significantly linked to improved adherence to PAP therapy (OR 1.096; *p* = 0.042). Additionally, longer rapid eye movement (REM) sleep during PAP titration (OR 1.112; *p* = 0.016) and a higher optimal pressure level (OR 1.261; *p* = 0.042) were significantly correlated with increased PAP adherence. Conversely, a lower ODI from PAP titration was significantly associated with improved adherence (OR 0.873; *p* = 0.003). Other demographic, polysomnographic, and PAP titration variables did not show significant associations.

A final logistic regression model of factors associated with short-term adherence is presented in Table 3. The logistic regression analysis was repeated with variables that showed significance in the preliminary model. Covariates included smoking status, ESS, RERA index from polysomnography, ODI, REM sleep, and optimal PAP level from PAP titration. Smoking status demonstrated a negative association with adherence, with smokers being significantly less likely to adhere compared to non-smokers (OR 0.267; 95% CI, 0.116–0.580; *p* = 0.001). The ESS exhibited a positive association with adherence, indicating that patients with higher daytime sleepiness were more likely to comply with PAP therapy (OR 1.080; 95% CI, 1.004–1.166; *p* = 0.042). Conversely, a higher ODI from PAP titration negatively impacted adherence, correlating with reduced adherence (OR 0.906; 95% CI, 0.829–0.975; *p* = 0.015). Moreover, a higher optimal pressure from PAP titration was positively associated with adherence, indicating that higher pressures increased the likelihood of adherence (OR 1.240; 95% CI, 1.007–1.119; *p* = 0.029).

**Figure 1:**
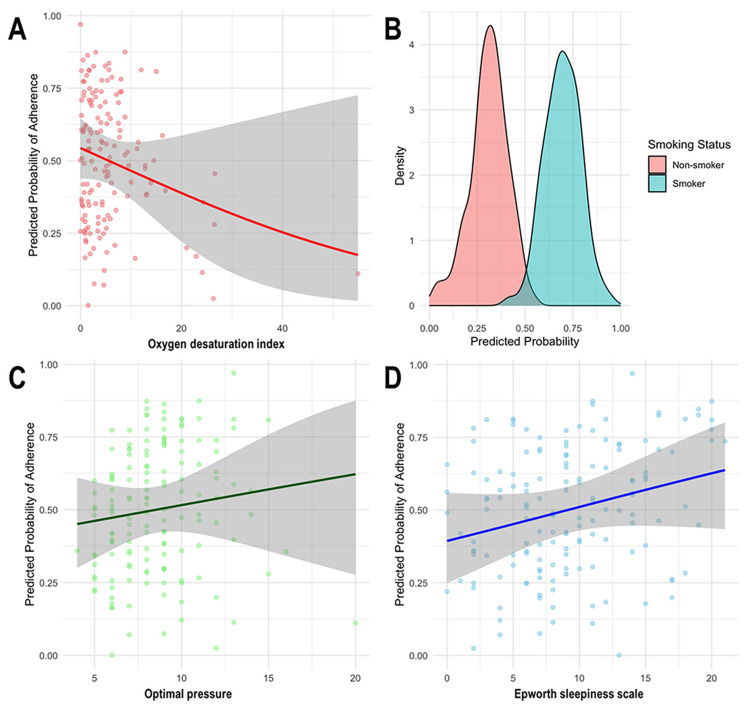
Associations between predicted probability of positive airway pressure (PAP) adherence and key clinical variables. This figure illustrates the relationship between predicted adherence to PAP therapy and four significant variables identified in the analysis. For continuous variables (Panels (**A**,**C**,**D**)), scatter plots are overlaid with smoothed regression lines and shaded 95% confidence intervals to show trends. For the categorical variable (Panel (**B**)), density plots are used to visualize group differences in predicted adherence probabilities. (**A**) Oxygen desaturation index (ODI) during PAP titration was negatively associated with adherence. A higher ODI was linked to a lower predicted probability of adherence. (**B**) Smoking status showed a clear group difference: smokers (blue) had a substantially lower predicted probability of adherence than non-smokers (pink). (**C**) Optimal pressure settings from PAP titration were positively associated with adherence, showing a mild upward trend. (**D**) Higher scores on the Epworth sleepiness scale (ESS), indicating greater daytime sleepiness, were associated with a higher probability of PAP adherence.

The predicted probability of short-term adherence to PAP therapy by significant factors, based on the final logistic regression model, is depicted in Figure 1. A negative association was noted between ODI from PAP titration and the predicted probability of adherence. The predicted probability of adherence was lower among smokers than non-smokers, as evidenced by distinct separations in the density distributions. Additionally, positive correlations were observed between optimal pressure from PAP titration and the predicted adherence probability and between ESS and the predicted probability of adherence.

**Figure 2:**
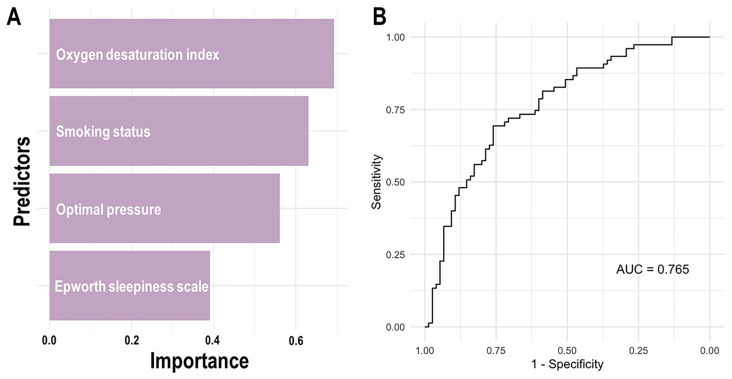
Variable importance and model performance for predicting positive airway pressure (PAP) adherence. (**A**) Among the predictors, the oxygen desaturation index from PAP titration emerged as the most significant, followed by smoking status, optimal pressure, and Epworth sleepiness scale, indicating their respective influences on adherence. (**B**) The receiver operating characteristic curve analysis revealed an area under the curve (AUC) of 0.765, suggesting the model’s effective discrimination ability in differentiating between adherent and non-adherent patients.

Variable importance and model performance for predicting short-term adherence to PAP therapy are demonstrated in Figure 2. ODI from PAP titration was identified as the most significant predictor, followed by smoking status, optimal pressure from PAP titration, and ESS. ROC curve analysis showed an AUC of 0.765, suggesting that the model effectively discriminates between patients who adhered to and those who did not adhere to PAP therapy.

## 4. Discussion

This study investigated factors influencing short-term PAP adherence by evaluating demographic and sleep study data across both adherence and non-adherence groups. The findings are summarized as follows: (1) The final logistic regression model indicated that smoking status, ESS, ODI from PAP titration, and optimal PAP levels were significantly associated with short-term adherence. Specifically, smokers were less likely to adhere, higher ESS scores and optimal PAP levels increased adherence, and a higher ODI from PAP titration decreased adherence; and (2) the AUC of 0.765, as indicated by the ROC curve analysis, demonstrates that the model effectively differentiates between adherence and non-adherence groups.

Smoking exacerbates sleep-disordered breathing by inducing airway inflammation, irritating mucosal linings, and increasing upper airway resistance [35]. Furthermore, multiple studies have reported that smoking reduces adherence to PAP therapy [36,37]. McArdle et al. [36] investigated the determinants of long-term PAP adherence in patients with sleep apnea/hypopnea syndrome by analyzing data from 1211 consecutive patients. They found that current smoking status was identified as a significant predictor of poor PAP adherence, with smokers being less likely to adhere to long-term PAP therapy compared to non-smokers [36]. Russo-Magno et al. [37], in a retrospective review of data from a Veterans Affairs Medical Center, identified factors influencing PAP compliance among 33 older male patients with OSA. Their findings indicated that cigarette smoking, nocturnal urination, and benign prostatic hypertrophy were significantly associated with non-adherence to PAP [37]. Consistent with these observations, the current study also showed that smoking adversely affects PAP adherence, thereby reducing the therapeutic efficacy of PAP treatment. Consequently, patients undergoing PAP therapy are strongly advised to cease smoking. Additionally, they should be referred to smoking cessation counseling and support programs to increase their chances of successful smoking cessation.

The potential mechanisms by which smoking may impair adherence to PAP therapy include the following: (1) Smoking induces inflammation and edema in the mucosal linings of both the upper and lower airways, which reduces airway diameter and increases resistance. These changes can lead to discomfort while using PAP, thereby reducing treatment adherence. (2) Smoking increases mucus secretion, which can more easily obstruct the airways. This obstruction may cause discomfort during PAP therapy due to disrupted airflow, ultimately reducing its effectiveness. (3) Patients experiencing difficulty breathing through their nose are less likely to adhere to PAP therapy. Smoking exacerbates nasal congestion and increases nasal airway resistance, potentially resulting in more significant discomfort while wearing a PAP mask. (4) Nicotine dependence can lead to withdrawal symptoms such as anxiety, irritability, and difficulty concentrating during overnight periods when smoking is not possible. These symptoms may decrease the persistence necessary for effective PAP therapy. (5) Smoking causes vasoconstriction, which reduces oxygen delivery to tissues. This effect lowers the elasticity of upper airway tissues, exacerbating airway collapse and diminishing the efficacy of PAP therapy. In addition, smoking has been associated with increased production of reactive oxygen species (ROS), leading to elevated oxidative stress. Oxidative stress can contribute to cellular damage and inflammatory responses, potentially impairing mucosal integrity and increasing airway sensitivity to positive pressure therapy. This heightened sensitivity may reduce patient comfort and, subsequently, hinder adherence to PAP treatment [38].

Numerous studies have demonstrated an association between the severity of OSA and adherence to PAP therapy [18,36]. Two primary methods are used to assess the severity of OSA: objective measurements and subjective evaluations. Objective measurements include respiratory parameters from polysomnography, such as the AHI and ODI, while subjective evaluations involve assessing symptoms such as daytime sleepiness through visual analog scales or questionnaires like ESS. This study confirmed significant associations between PAP adherence and factors such as the ODI from PAP titration, optimal pressure, and daytime sleepiness as measured by the ESS, all closely linked to the severity of OSA. The analysis demonstrated that higher ESS scores and elevated optimal pressure levels were positively correlated with improved adherence to PAP therapy. This suggests that patients experiencing greater daytime sleepiness or requiring higher optimal therapeutic pressure levels due to the severity of their disease may perceive greater benefits from consistent usage. Evidence from multiple clinical studies indicates that as OSA severity based on objective measurements increases, the corresponding optimal PAP pressure also rises [39]. Conversely, a higher ODI measured from PAP titration was associated with lower PAP adherence, a phenomenon potentially explained by the discomfort or persistent respiratory disturbances caused by frequent oxygen desaturation events, even though PAP devices were used. More specifically, the ODI during PAP titration emerged as the single best predictor of treatment adherence, likely because it more accurately reflects the physiological impact of OSA than traditional indices like the AHI [40]. Intermittent hypoxia, as captured by ODI, contributes to systemic effects such as sympathetic activation and inflammation, which are closely linked to patient symptoms and health outcomes. Therefore, stabilization of oxygen saturation during titration may enhance patients’ perception of PAP therapy effectiveness, improving adherence and highlighting ODI as a clinically valuable predictor. Meanwhile, in this study, the RERA index measured during PAP titration showed a negative correlation with therapy adherence. This finding suggests that, similar to the oxygen desaturation index (ODI), a reduction in RERA through appropriate PAP titration may contribute to improved adherence. By contrast, the total arousal index measured during baseline polysomnography did not emerge as a significant predictor. However, patients with a low arousal threshold or a high frequency of RERAs are generally expected to have more difficulty tolerating PAP therapy, which may result in lower adherence. From this perspective, patients with a high RERA index on baseline polysomnography may be at increased risk of non-adherence, and this warrants further clinical consideration.

In this study, the primary predictor of short-term adherence to PAP therapy was the ODI measured during PAP titration, followed by factors such as smoking status, optimal PAP pressure, and the ESS. The importance of these variables is determined by the standardized beta coefficients from the final logistic regression model. These coefficients facilitate the comparison of variables measured on different scales by neutralizing the impact of their units. This method allows for an assessment of each predictor’s relative contribution to the dependent variable, in this case, short-term adherence to PAP therapy. The predictive performance of the final model, evaluated using ROC curve analysis, resulted in an AUC of 0.765, indicating effective discrimination between the adherent and non-adherent groups. While this demonstrates acceptable predictive accuracy, there is room for further model optimization. Enhancements such as increasing the sample size, refining feature selection, or incorporating additional predictors could enhance its discriminative capability. The AUC value highlights the model’s potential utility in clinical settings to identify patients at risk of non-adherence.

Short-term adherence was emphasized in this study not only due to design considerations but also because it represents a clinically significant concern. Previous studies have consistently shown that early adherence patterns are strong predictors of long-term compliance with PAP therapy [41]. Specifically, Van Ryswyk et al. [41] demonstrated a significant association between adherence in the first few weeks to months and long-term adherence at 24 months, suggesting that the initial treatment period is critical for the establishment of sustained usage behaviors. Additionally, earlier research has identified factors such as high therapeutic pressure and symptom severity (e.g., severe snoring) as important determinants of adherence, which further emphasizes the importance of early-phase adaptation [42,43]. These findings support the idea that timely identification of barriers and facilitators during the early stages of therapy may be essential for optimizing long-term treatment outcomes. In this context, our study was designed to explore factors influencing short-term adherence from the planning stage, with the broader aim of informing future strategies to enhance long-term adherence. The insights gained here may serve as a foundation for the development of early interventions tailored to improve both immediate and sustained engagement with PAP therapy.

This study has several limitations. First, its retrospective design may affect the interpretation of the results. Second, by adopting the Korean National Health Insurance criteria for PAP adherence—which specifies 30 days of usage within the initial 90 days of treatment—our study’s findings may have limited generalizability to other healthcare settings, despite aligning with international usage thresholds. Third, smoking status was not included as a matching variable in the propensity score matching process, which may have introduced residual confounding between groups. Fourth, this study focused only on short-term adherence, which may not fully represent patients’ long-term compliance with PAP therapy. Future research should aim to explore long-term adherence patterns and validate these findings across diverse clinical settings and patient populations.

## 5. Conclusions

The findings of this study highlight several key predictors of short-term adherence to PAP therapy, offering valuable guidance for clinical practice and future interventions. Notably, smoking status showed a strong negative correlation with adherence, suggesting that smoking cessation support should be integrated into initial treatment planning. Daytime sleepiness and the quality of PAP titration also emerged as important factors, underscoring the need for individualized pressure settings and early symptom assessment.

From a clinical standpoint, these results support three practical recommendations: (1) proactively addressing smoking behavior at the time of OSA diagnosis; (2) incorporating validated tools such as the Epworth sleepiness scale to identify patients at risk of poor adherence; and (3) optimizing patient comfort through early, individualized PAP titration. Implementing these strategies may enhance early adherence, which is closely linked to long-term treatment success. Future studies should explore how these predictors can inform personalized adherence programs to further improve outcomes in patients with OSA.

## Figures and Tables

**Table 1:** Demographic and sleep study data between adherence and non-adherence groups (*n* = 150).

Characteristics	Adherence Group (*n* = 75)	Non-adherence Group (*n* = 75)
Demographic data		
Age (years)	49.91 ± 14.25	49.21 ± 14.47
Gender (male:female)	60:15	59:16
Body mass index (kg/m^2^)	29.09 ± 4.69	28.83 ± 5.54
Smoking status (yes:no)	35:40	58:17
ESS	9.75 ± 5.06	8.55 ± 5.06
PSQI	5.81 ± 2.45	5.36 ± 2.23
Polysomnographic data		
TST (minutes)	341.64 ± 42.73	344.93 ± 45.25
Sleep efficiency (%)	81.69 ± 11.86	81.07 ± 12.34
Respiratory arousal index	32.79 ± 22.32	27.32 ± 23.19
Spontaneous arousal index	5.95 ± 10.18	5.14 ± 5.30
Total arousal index	42.13 ± 19.93	38.10 ± 20.85
Stage N1 (% of TST)	28.87 ± 17.16	25.67 ± 14.60
Stage N2 (% of TST)	42.38 ± 15.65	45.54 ± 14.81
Stage N3 (slow-wave sleep) (% of TST)	4.89 ± 6.65	4.25 ± 5.73
Stage R (REM sleep) (% of TST)	15.91 ± 6.11	16.08 ± 5.47
AHI (events/hour of TST)	43.56 ± 23.19	37.26 ± 24.06
Supine AHI	52.31 ± 24.55	47.84 ± 27.18
RERA index	3.84 ± 3.13	5.98 ± 7.70
RDI (events/hour of TST)	47.39 ± 21.55	42.58 ± 22.31
ODI (events/hour of TST)	40.59 ± 23.52	36.29 ± 23.95
PAP titration data		
TST (minutes)	356.65 ± 43.02	347.81 ± 51.09
Sleep efficiency (%)	84.57 ± 9.66	81.31 ± 13.39
Total arousal index	19.82 ± 10.31	20.64 ± 12.78
Stage N1 (% of TST)	15.01 ± 8.89	15.20 ± 8.84
Stage N2 (% of TST)	51.10 ± 13.05	50.58 ± 12.76
Stage N3 (slow-wave sleep) (% of TST)	7.14 ± 7.26	6.60 ± 7.54
Stage R (REM sleep) (% of TST)	20.69 ± 5.76	17.96 ± 7.43
AHI (events/hour of TST)	5.27 ± 5.62	7.19 ± 9.91
Supine AHI	5.52 ± 5.76	7.24 ± 9.69
RERA index	1.43 ± 1.60	1.31 ± 1.42
RDI (events/hour of TST)	6.68 ± 5.49	8.48 ± 10.00
ODI (events/hour of TST)	4.91 ± 4.76	6.33 ± 8.64
Optimal pressure	8.69 ± 2.34	8.40 ± 2.87

Data are presented as mean ± standard deviation. ESS, Epworth sleepiness scale; PSQI, Pittsburgh sleep quality index; TST, total sleep time; N, non-rapid eye movement; REM, rapid eye movement; AHI, apnea–hypopnea index; RERA, respiratory effort-related arousal; RDI, respiratory disturbance index; ODI, oxygen desaturation index; PAP, positive airway pressure.

**Table 2:** Initial logistic regression model of factors associated with short-term adherence.

Dependent Variable	Independent Variables	B	SE	OR	95% CI	*p*
Adherence	(Intercept)	−7.815	3.567	0.000	0.000–0.439	0.028 *
	Smoking status	−1.200	0.456	0.301	0.123–0.736	0.009 **
	ESS	0.091	0.045	1.096	1.004–1.198	0.042 *
	PSQI	0.127	0.091	1.135	0.949–1.357	0.164
	TST ^†^	−0.005	0.009	0.995	0.977–1.012	0.552
	Sleep efficiency ^†^	0.037	0.034	1.038	0.970–1.111	0.277
	Spontaneous ArI ^†^	0.037	0.031	1.038	0.976–1.103	0.233
	N1 sleep ^†^	0.013	0.022	1.013	0.970–1.057	0.557
	N2 sleep ^†^	−0.006	0.025	0.994	0.947–1.043	0.808
	N3 sleep ^†^	0. 063	0.044	1.066	0.978–1.161	0.147
	REM sleep ^†^	0.007	0.040	1.007	0.931–1.090	0.858
	RERA index ^†^	−0.162	0.068	0.851	0.745–0.971	0.017 *
	TST ^‡^	0.002	0.006	1.002	0.990–1.014	0.733
	Sleep efficiency ^‡^	−0.023	0.029	0.976	0.992–1.034	0.412
	Total ArI ^‡^	0.061	0.038	1.063	0.988–1.145	0.102
	N1 sleep ^‡^	0.007	0. 039	1.007	0.932–1.145	0.851
	N2 sleep ^‡^	0.037	0.027	1.038	0.983–1.095	0.176
	N3 sleep ^‡^	0.032	0.041	1.032	0.953–1.118	0.433
	REM sleep ^‡^	0.106	0.044	1.112	1.020–1.213	0.016 *
	ODI ^‡^	−0.136	0.046	0.873	0.798–0.956	0.003 **
	Optimal pressure	0.232	0.114	1.261	1.008–1.577	0.042 *

ESS, Epworth sleepiness scale; PSQI, Pittsburgh sleep quality index; TST, total sleep time; ArI, arousal index; N, non-rapid eye movement; REM, rapid eye movement; RERA, respiratory effort-related arousal; ODI, oxygen desaturation index; SE, standard error; OR, odds ratio; CI, confidence interval. * *p* < 0.05; ** *p* < 0.01; ^†^ polysomnography; ^‡^ positive airway pressure titration.

**Table 3:** Final logistic regression model of factors associated with short-term adherence.

Dependent Variable	Independent Variables	B	SE	OR	95% CI	*p*
Adherence	(Intercept)	−1.95	1.018	0.142	0.018–1.003	0.055
	Smoking status	−1.32	0.407	0.267	0.116–0.580	0.001 **
	ESS	0.08	0.038	1.080	1.004–1.166	0.042 *
	RERA index ^†^	−0.11	0.056	0.900	0.802–0.994	0.057
	ODI ^‡^	−0.10	0.041	0.906	0.829–0.975	0.015 *
	REM sleep ^‡^	0.05	0.028	1.048	0.992–1.109	0.097
	Optimal pressure ^‡^	0.21	0.099	1.240	1.007–1.119	0.029 *

ESS, Epworth sleepiness scale; RERA, respiratory effort related arousal; ODI, oxygen desaturation index; REM, rapid eye movement; SE, standard error; OR, odds ratio; CI, confidence interval. * *p* < 0.05; ** *p* < 0.01; ^†^ polysomnography; ^‡^ positive airway pressure titration.

## Data Availability

The datasets used and/or analyzed during the current study may be provided by the corresponding author, upon appropriate request.

## Abbreviations

The following abbreviations are used in this manuscript:

OSA	Obstructive sleep apnea
PAP	Positive airway pressure
AHI	Apnea–hypopnea index
BMI	Body mass index
ESS	Epworth sleepiness scale
PSQI	Pittsburgh sleep quality index
TST	Total sleep time
SE	Sleep efficiency
RERA	Respiratory effort-related arousal
RDI	Respiratory disturbance index
ODI	Oxygen desaturation index
ORs	Odds ratios
CIs	Confidence intervals
ROC	Receiver operating characteristic
AUC	Area under the curve
REM	Rapid eye movement
SDB	Sleep-disordered breathing
